# Diversification of *Pseudomonas aeruginosa* After Inhaled Tobramycin Therapy of Cystic Fibrosis Patients: Genotypic and Phenotypic Characteristics of Paired Pre- and Post-Treatment Isolates

**DOI:** 10.3390/microorganisms13040730

**Published:** 2025-03-24

**Authors:** Dayana Borisova, Tanya Strateva, Svetoslav G. Dimov, Borjana Atanassova, Tsvetelina Paunova-Krasteva, Tanya Topouzova-Hristova, Svetla T. Danova, Rositsa Tropcheva, Stoyanka Stoitsova

**Affiliations:** 1 The Stephan Angeloff Institute of Microbiology, Bulgarian Academy of Sciences, Acad. G. Bonchev Str., Bl. 25, 1113 Sofia, Bulgaria; daqanara@abv.bg (D.B.); pauny@abv.bg (T.P.-K.); stdanova@yahoo.com (S.T.D.); 2 Department of Medical Microbiology “Corr. Mem. Prof. Ivan Mitov, MD, DMSc”, Faculty of Medicine, Medical University of Sofia, 2 Zdrave Str., 1431 Sofia, Bulgaria; dr.strateva@abv.bg; 3 Faculty of Biology, Sofia University “St. Kliment Ohridski”, 8 Dragan Tsankov Blvd., 1164 Sofia, Bulgaria; svetoslav@biofac.uni-sofia.bg (S.G.D.); borjana.atanassova@abv.bg (B.A.); topouzova@biofac.uni-sofia.bg (T.T.-H.); 4 Center of Applied Studies and Innovation, 8, Dragan Tsankov Blvd., 1164 Sofia, Bulgaria; rositsa.tropcheva@casi.bg

**Keywords:** *Pseudomonas aeruginosa*, paired cystic fibrosis pre- and post- treatment isolates, inhaled tobramycin treatment, RAPD-PCR, growth, biofilm, invasion in A549 cells, sub-lethal amounts of tobramycin

## Abstract

This study examines the impact of inhaled tobramycin therapy on the within-host changes in *P. aeruginosa* strains isolated from Bulgarian patients with CF prior to and post treatment. Genotypic comparison by RAPD-PCR indicated that most of the pre-treatment isolates had a high similarity and were genetically comparatively close to strains from other countries with known increased morbidity or treatment requirements. Most of the post-treatment isolates were, however, genetically distant from their pre-treatment counterparts, showing genotypic diversification after the treatment. Phenotypic comparisons showed a lower ODmax reached during groswth and an increased lag-time in the post-treatment isolates. All strains were capable of invasion and intracellular reproduction within A549 cultured cells. The addition of sub-inhibitory amounts (1/4 or 1/2 MIC) of tobramycin during growth showed the higher relative fitness (as a percentage of the untreated control) of the post-treatment strains. The effects of sub-MICs on biofilm growth did not show such a pronounced trend. However, when a resazurin-based viability test was applied, the advantage of the post-treatment strains was confirmed for both broth and biofilm cultures. In spite of that, according to the determined MIC values, all isolates were tobramycin-sensitive, and the data from this study imply the development of tolerance to the antibiotic in the strains that survived the treatment.

## 1. Introduction

Cystic fibrosis (CF) is an autosomal-recessive disorder related to mutations of the *CFTR* gene that is responsible for the synthesis of an epithelial ionic channel. In the homozygotic state, the mutations result in a reduction in the secretion of chloride ions and, hence, of the pH. As a consequence, thick mucus together with entrapped bacteria accumulate in CF lungs. The effectiveness of the innate defense mechanisms is reduced in the altered CF lung microenvironment, and this promotes the establishment and chronification of bacterial infections. The latter are the major causes of exacerbation and death of the patients [1,2,3].

Among the opportunistic bacteria which settle in CF airways, *Pseudomonas aeruginosa* has a leading role. Within the host lungs, it undergoes a series of genotypic and phenotypic changes under the selective pressure of the microenvironment. This results in the formation of well-adapted strains.

Efforts to overcome the disease have lately been directed at both the CF mechanisms themselves and the accompanying bacterial infections. Successes in the development of CFTR modulators applicable either alone or in combination with antibiotics [4] deserve mentioning, as well as recent research advances in gene therapy [5]. Among the novel antibacterial strategies, the potential of phage therapies is very promising [6,7], with even some initial data for cases of successful clinical applications, e.g., in patients with pan-drug-resistant *P. aeruginosa* [8]. However, the wide-scale application of phage therapy still poses lots of various unsolved problems [6,9]. Therefore, antibiotic therapy is still the dominating approach of choice.

Tobramycin, an aminoglycoside antibiotic which binds to the 30S ribosomal subunit and blocks bacterial protein synthesis [10], is widely used for the treatment of Gram-negative infections in patients with CF. It is particularly useful against *P. aeruginosa* because of the lower rates of resistance of clinical isolates [11]. Its application via inhaled preparations ensures reliable delivery directly to the infected loci in the lungs, with positive clinical outcomes in terms of efficiency and safety [12,13]. Nevertheless, under the pressure of the immune system of the host and the therapy itself, the bacteria undergo adaptive changes, and chronic infections by this pathogen are virtually impossible to eradicate with the antibiotic treatment [3].

*P. aeruginosa* can evade both the defense mechanisms of the CF host and therapy for decades; therefore, the colonization of CF lungs by this microorganism provides a unique opportunity to investigate the pathogen’s evolution over time [14]. Over the course of the routine control of patients, serial isolates are collected. The comparative genotypic and phenotypic investigations of such strains provide valuable data on the within-host evolution of *P. aeruginosa* [3,15,16]. Such studies contribute greatly not only to the understanding of the within-host evolution of *P. aeruginosa* but also to the more general notion of the switch from acute to chronic bacterial diseases [17].

During infections, genetic heterogeneity arises due to mutations in the *P. aeruginosa* population [18,19]. The pressure of the factors present in CF lungs (antibiotics, immune mediators, iron deficiency, mucus, reduced oxygen availability) results in the in-host selection of specific phenotypes. The latter, while reducing the fitness of the bacteria in other media, provide the strains with advantages for survival in the specific CF microenvironment. The adaptive within-host selection among the diverse genotypes results in the establishment of more or less convergent phenotypes, frequently occurring among serial *P. aeruginosa* isolates [16,20]. Mutations in CF strains are often linked to metabolic functions, quorum-sensing regulation, virulence-associated genes, and also genes that shape a phenotypic conversion of *P. aeruginosa*, e.g., *LPS* deficiency, enhanced biofilm formation, alginate production, etc. [16,21,22]. The evolutionary adaptations include the reduced production of virulence factors, slow growth, transition to a biofilm-associated mode of life, selection for antibiotic tolerance or resistance, mucoidity, reduction in motility, and occurrence of auxotrophy. Notably, the selection of such phenotypes has been shown to occur repeatedly and independently across multiple patients, suggesting a strong influence of the CF environment [3]. Genotypic and phenotypic differences have been shown to be related to the time of patient colonization, and differences have been noted between early, intermediate, and late isolates [16,19,23,24].

Once *P. aeruginosa* has been established permanently in CF airways, chemotherapy may diminish the bacterial load, reduce inflammation, and improve lung function, but it is virtually impossible to eradicate the infection [3,19]. The development of novel chemotherapeutic approaches has permitted the improvement of therapy and has increased in the life span of patients with CF. Systemic (intravenous and oral) antibiotics are still routinely applied, but their efficiency is reduced due to the formation of bacterial biofilms. Therefore, the therapy employing inhaled antibiotics is presently favored [10]. Despite the advances in control procedures, infection therapy is not entirely effective [1].

Together with the specific factors of the CF microenvironment, the within-host selection and evolution of bacteria is undoubtedly influenced by the patient’s regular antibiotic treatment. However, the role of antibiotics as in vivo evolutionary factors is still insufficiently explored, with only a few studies directly addressing some antibiotic-driven phenotypic shifts. One study on *P. aeruginosa* strains isolated from sputum samples pre and post intravenous treatment with meropenem/colistin indicated the occurrence of phenotypic fluctuations between the isolates [25]. Another study on paired strains collected shortly before and after suppressive therapy (intravenous application of combinations of two antibiotics plus inhaled antibiotics) was focused on antibiotic resistance patterns, mucoidity, biofilm presence in sputum, and hypermutability [26].

The objectives of the present study were to trace some genotypic and phenotypic differences between CF paired clinical strains that could have possibly developed as a consequence of inhaled tobramycin application in patients.

To address this, we performed a genotypic and phenotypic comparison of six pairs of strains from six Bulgarian patients, each pair comprising one pre-treatment and one post-inhaled tobramycin application isolate from the same patient. The molecular epidemiology part of this study aimed to compare the results between the twelve Bulgarian isolates in focus, as well as between them and several CF transmissible or serial isolates originating from different geographic loci throughout the world, included in the international reference panel [27]. The phenotypic comparisons included some widely examined phenotypes like growth parameters, biofilm formation, invasion in cultured epithelial cells, and motility.

Next, we addressed the question of whether the inhaled tobramycin treatment influenced the response of the post-treatment strains to this antibiotic. First, the MICs of tobramycin of the isolates were determined. Then, we searched for the occurrence of possible shifts in some fitness parameters (growth, biofilm, viability, and motility) of the pre- and post-treatment isolates when they were cultivated in the presence of sub-lethal amounts (quarter or half MIC) of tobramycin. Since the CF lung microenvironment is characterized by a spatial heterogeneity and an obstructed penetration of antibiotics through the host sputum, thus generating intrapulmonary gradients, sub-inhibitory concentrations of antibiotics occur in these patients [28,29,30], justifying the study of their effects in our comparative experiments.

## 2. Materials and Methods

### 2.1. Bacterial Strains and Culture Media

Twelve *P. aeruginosa* clinical strains (six pairs) were included in this study. The paired strains were collected during 2006–2009. They were obtained from sputum samples of Bulgarian patients with CF during routine laboratory diagnostics in the Department of Medical Microbiology of the Medical University of Sofia. The isolation took place prior to and post treatment of the patients with 2 to 15 cycles of inhaled tobramycin (Table 1). Species identification was performed using the BD Phoenix M50 automated system (BD, Franklin Lakes, NJ, USA).

All procedures were performed in accordance with the ethical principles for medical research involving human participants of the Helsinki Declaration of 1964 and its later amendments (https://www.wma.net/what-we-do/medical-ethics/declaration-of-helsinki/, accessed on 8 March 2025). This study involved solely the bacterial isolates, and no personal patient information was used.

The molecular epidemiology typing studies also included the following *P. aeruginosa* strains from the international reference panel [27]: paired or sequential isolates (AA2, AA43, AA44; AMT0023-30, AMT0023-34; AMT0060-1, AMT0060-2 1nd AMT0060-3); CF transmissible strains (LESB58, LES431, C3719, AES-1R and AUS23); and commonly used strains PAO1 and CHA. These strains are available from BCCM/LMG Bacteria Collection, Ghent University, Gent, Belgium (http://bccm.belspo.be/about-us/bccm-lmg, accessed on 10 January 2025). In addition, PAO1 was used as a reference strain throughout all experiments in this study. All strains were maintained as frozen stock at −80 °C, with 8% DMSO as the cryoprotector.

The culture media used were tryptic soy broth (TSB) and agar (TSA) (Sigma-Aldrich, St. Louis, MO, USA), Mueller-Hinton broth (MHB) (Sigma-Aldrich, St. Louis, MO, USA), and minimal salt medium M63 (0.02 M KH_2_PO_4_, 0.04 M K_2_HPO_4_, 0.02 (NH_4_)_2_SO_4_, 0.1 mM MgSO_4_, and 0.04 M glucose). Where appropriate, the liquid media were solidified with agar (Difco, Thermo Fisher Scientific, Waltham, MA, USA).

Before the experiments, samples from the frozen stocks were streaked on TSA plates and single colonies were inoculated in TSB and cultivated overnight at 37 °C. Then, the bacteria were applied onto slanted agar, cultivated overnight and kept refrigerated at 0–4 °C as sources of inoculum, for no more than 25 days.

### 2.2. Random Amplification of Polymorphic DNA (RAPD)–PCR Analysis

RAPD-PCR with a single arbitrary primer was used to check the genetic relatedness between the paired isolates and between them and CF strains of different geographic origin, included in the international reference panel [27]. Genomic DNA of the strains was isolated with the HigherPurity™ Bacterial Genomic DNA Isolation Kit (Canvax Biotech, Saint Louis, MO, USA) from overnight bacterial cultures, following the manufacturer’s protocol. The DNA concentration and purity were determined by absorbance readings at 260/280 nm (NanoDrop 1000, Thermo Scientific, Waltham, MA, USA). RAPD was performed with primer 272 (5′-AGCGGGCCAA-3′) [31]. The analysis was performed using Ready-To-Go RAPD Analysis Beads (GE Healthcare, Sofia, Bulgaria). PCR amplification was conducted in accordance with the manufacturer’s instructions: A reaction mixture was prepared containing 12.5 µL Mastermix (Thermo Scientific, Waltham, MA, USA), 3 µL of primer 272 (10 pmol), 1, 5 mL of DNA, and H_2_O, to a final volume of 25 µL. Amplifications were carried out in an Eppendorf Gradient Mastercycler (Eppendorf, Hamburg, Germany) as follows: 5 min at 94 °C; 4 cycles of 5 min at 36 °C, 5 min at 72 °C, and 5 min at 94 °C; 30 cycles of 1 min at 94 °C, 1 min at 36 °C, and 2 min at 72 °C; and a 10 min hold at 72 °C. RAPD–PCR products were applied on 2% agarose gel, with 100 bp DNA-ladder (Canvax Biotech, Saint Louis, MO, USA), and they were run at 3.5 V for 4 h.

### 2.3. Unweighted Pair–Group Method with Arithmetic Mean (UPGMA) Analysis

Polymorphic RAPD patterns of the studied CF *P. aeruginosa* isolates were subjected to UPGMA analysis. The software GeneTools v.4.1 (Syngene, Cambridge, UK) was used for similarity matrix calculations and the construction of UPGMA dendrograms using the “profile” and “band position” options. The dendrograms were based on the matrices of the similarity coefficients. A similarity of >70% was used as a threshold for clonal relatedness of the strains [32].

### 2.4. Growth Curve Analysis

For the preparation of the inoculum, from the slanted agar cultures, 18 h broth cultures were prepared in TSB. Then, 50 µL of the bacteria was inoculated in 5 mL of MHB or M63 media, vortexed, and distributed into the wells of 96-well U-bottomed microreader plates (Corning, New York, NY, USA). Each well contained 150 µL bacterial suspension, with 6 repeats per strain. The samples were cultivated at 37 °C for 48 h. The bacterial growth was monitored by measuring hourly the optical density at 620 nm (OD_620nm_) on a plate reader LTEK INNO (LTEK, Gyeonggi-do, Republic of Korea). Prior to each measurement, the plates were shaken on the IKA s Basic shaker (IKA-Werke, Staufen, Germany) for 2 min, at 5000 rpm. *P. aeruginosa* PAO1 was used as a reference strain. The OD_max_ values and the lag-times were determined from the growth curves.

### 2.5. Biofilm-Growth Evaluation

The biofilm growth was evaluated by the crystal violet method [33]. The biofilms were cultivated in MHB or M63 media on 96-well polystyrene U-bottomed plates (Corning, Corning, NY, USA), inoculated as above. *P. aeruginosa* PAO1 was used as a reference strain. The biofilms were cultivated at 37 °C for 24 h. Plankton bacterial cells were removed, the plates were washed in 3 changes of phosphate buffered saline (PBS) and the attached cells were colored for 15 min with 0.1% *w*/*v* aqueous crystal violet (CV). Then, the wells were thoroughly washed with several changes of PBS until complete disappearance of the bluish color in the pipette tips. This was followed by the solubilization of the dye in 70% ethanol for 15 min with continuous shaking. The absorbance was measured on a plate reader LTEK INNO (LTEK, Gyeonggi-do, Republic of Korea) at 595 nm wavelength.

### 2.6. Invasion of A549 Cells

The A549 cell line (NBIMCC, Sofia, Bulgaria) was cultivated at 37 °C, 5% CO_2_ in DMEM (Sigma-Aldrich, St. Louis, MO, USA) with the addition of 10% FBS (Sigma-Aldrich, St. Louis, MO, USA) and 1% antibiotic/antimycotic supplement (VWR international bv, Leuven, Belgium) (containing 100 U mL^−1^ penicillin, 100 µg mL^−1^ streptomycin, and 0.25 µg mL^−1^ amphotericin B). The co-cultivation of the cells with the bacteria followed the protocol earlier described [34]. For co-cultivation with the bacteria, the cells were cultivated in 25 mL TTP flasks (Orange Scientific, Geneneva, Switzerland, Europe) to 80–100% confluence. Then, the culture medium was removed, and the cell monolayers were gently washed with three changes of sterile PBS for removal of the antibacterials. Overnight TSB cultures from the bacterial strains were pelleted, washed in PBS, and calibrated to 0.5 McFarland units (10^9^ cells mL^−1^) using Densilameter II (Erba Lachema, Brno, Czech Republic). The microorganisms were diluted further in DMEM/10% FBS without the antibacterial supplement to 10^5^ cells mL^−1^ and applied to the A549 culture at an MOI of approximately 0.25. The co-cultivation was performed for 2 h at 37 °C, 5% CO_2_. Then, the culture medium with the bacteria was removed, and fresh DMEM supplemented with 10% FBS and 1% antibiotic/antimycotic mixture, containing additional 10 µg mL^−1^ tobramycin (Sigma-Aldrich, St. Louis, MO, USA), was added for the elimination of extracellular bacteria. The cells were cultivated for a further 24 h. The presence/absence of extracellular bacteria in the medium was monitored by inoculating 50 µL of the culture medium in 5 mL of TSB, followed by 24 h cultivation at 37 °C. Then, the monolayers were washed in 3 changes of PBS, and the A549 cells were permeabilized with 0.2% Tween-20 (Sigma-Aldrich, St. Louis, MO, USA) in PBS for 15 min. The released bacteria were pooled, serially diluted, and plated on TSA plates for CFU enumeration.

### 2.7. Fluorescence Microscopy

For visualization of the possible intracellular localization of the invading bacteria, the A549 cells were cultivated on cover glasses placed in the wells of a 24-well plate. Cell cultivation and co-cultivation with bacteria were carried out as described in Section 2.6. After 2 h of co-cultivation in the absence of antibiotics and 24 h cultivation in the presence of antibacterials, as described in Section 2.6, the cells were washed with PBS containing 133 µg mL^−1^ Ca++ and 100 µg mL^−1^ Mg++ (PBS/Ca, Mg), fixed for 20 min with 4% buffered formalin and washed with PBS/Ca, Mg overnight, at 4 °C, to remove the extra fixative. The samples were then permeabilized for 20 min with 0.2% Tween-20. The cell nuclei and the bacteria were visualized by SYBR Green (Sigma-Aldrich, St. Louis, MO, USA). The actin cytoskeleton was labeled with 0.3 µg mL^−1^ TRITC-phalloidin (Sigma-Aldrich, St. Louis, MO, USA) [34]. The samples were embedded in Fluoromount^tm^ Aqueous Mounting Medium (Sigma-Aldrich, St. Louis, MO, USA). The observations were made on Nikon Eclipse TiU-1 using green and blue light filters. The images were registered with a Nikon DS-Fi1 (Nikon, Melville, NY, USA) camera and the NIS (Ver. 4.0) elements software.

### 2.8. Motility Assays

Motility assays were performed following the protocols described by Cullen et al. [33]. For the assays, TSB was solidified with different amounts of agar (Difco, Thermo Fisher Scientific, Waltham, MA, USA). For swimming motility, 0.3% *w*/*v* agar plates were used. For swimming motility 0.3% *w*/*v* agar plates were 280 used. with TSB supplemented with 0.02% glucose. Overnight 18 h bacterial cultures in TSB were used as the inoculum. The motility plates were inoculated at the center of the plate using sterile toothpicks. All motility plates were situated horizontally and incubated for 24 h at 37 °C. Each sample was repeated in triplicate. Two perpendicular measures of the diameters of the motility zones were taken. Twitching motility tests were performed on 1% TSA, also in triplicates per sample. The bacteria were inoculated by stabbing down to the bottom of the Petri dish. Following 24 h incubation at 37 °C, the agar was removed, and the bacterial twitching zones were visualized by means of 0.1% crystal violet.

### 2.9. Determining the Tobramycin Susceptibility of the Strains

The MICs of tobramycin of the strains were evaluated by means of graduated test-strips for tobramycin sensitivity (Himedia, Maharashtra, India) following the producer’s instructions. Briefly, the strains were cultivated for 18 h in MHB, then calibrated to 0.5 Mac Farland units (1 × 10^9^ cells/mL) using Densilameter II (Erba Lachema, Brno, Czech Republic). Then, 100 μL of the suspensions was spread onto MHA plates. Test-strips containing a concentration gradient of tobramycin were placed on the surface of each plate, and the samples were cultivated for 18 h at 37 °C. The results were read using the scale of the test-strips and interpreted according to the European Committee on Antimicrobial Susceptibility Testing (EUCAST)’s recommendations [35].

### 2.10. Phenotypic Tests on the Effects of Sub-MICs of Tobramycin

To check whether the in-host treatment with inhaled tobramycin influenced tolerance to the drug, experiments directed at the effects of sub-MIC of the antibiotic were undertaken. For this purpose, the above-described methodologies for characterizing bacterial phenotypic characteristics (growth, biofilm formation, and motility) were carried out in the presence of the quarter and half MIC amounts determined for each strain.

The effects of quarter and half MIC on bacterial growth and biofilm formation were tested on MHB and M63 media supplemented with the respective amounts of tobramycin (Sigma-Aldrich, St. Louis, MO, USA). For each strain, 6 wells containing each of the two sub-MIC amounts plus 6 control wells (the growth media without antibiotic) were included. Bacterial growth and biofilm formation followed the protocols described in Section 2.4 and Section 2.5. The results were calculated as the % of the untreated control values of, respectively, the ODmax_620nm_ for bacterial growth in the absence of the antibiotic and the A_595nm_ after crystal violet staining for the bacterial biofilms.

The experiments on bacterial motility were performed according to the methodologies described in Section 2.8, with the addition of quarter or half amounts of tobramycin determined for each of the strains to the motility agars.

### 2.11. Viability of Bacteria Cultivated in the Presence of Quarter or Half MIC of Tobramycin

The viability shifts in the presence of the antibiotic were evaluated by the resazurin-based viability test, using the Alamar Blue Cell Viability Reagent (Invitrogen, Thermo Fisher Scientific, Waltham, MA, USA), as described in [36]. For this set of experiments, growth and biofilm formation were tested on M63 medium, in the absence (controls) or presence of quarter or half of the MICs, determined for each individual strain. Each variant was repeated in 6 wells. For bacteria grown in broth, the M63 medium was supplemented with 1% *v*/*v* of Alamar Blue reagent, and the absorbance of the reduced (A_570_) and oxidized (A_600_) dyes was measured at hour 24. For biofilm metabolic activities, 24 h biofilms were first cultivated in the absence (control) or presence of the antibiotic; then, the unattached bacteria were removed, and the wells were washed in sterile PBS, changing the medium three times. Then, the wells were filled with 150 µL of M63 medium not supplemented (control) or supplemented with the respective amounts of the antibiotic, containing 1% *v*/*v* of the reagent, and incubated for a further 24 h. A_570_ and A_600_ were measured at hour 24.

The results for each strain and treatment were calculated as a percentage of the corresponding untreated control according to the following formula:

Viability (% of control) = [(ε_ox_)_λ2_A_λ1_ − (ε_ox_)λ_1_A_λ2_]/[(ε_ox_)_λ2_A’_λ1_ − (ε_ox_)_λ1_A’_λ2_] × 100

where A_λ1_ and A_λ2_ are the absorbance values measured at λ1 = 570 nm and λ2 = 600 nm for the test samples grown in the presence of the sub-MICs, and A’_λ1_ and A’_λ2_ are the absorbance values measured for the controls at λ1 = 570 nm and λ2 = 600 nm. The values of the extinction molar coefficients of the Alamar Blue reagent ((ε_ox_)_570_ = 80.586 and (ε_ox_)_600_ = 117.216), as well as the calculation methodology, followed the manufacturer’s instructions.

### 2.12. Scanning Electron Microscopy (SEM)

Flat polystyrene probes, 0.5 × 0.5 cm, were cleaned with detergent followed by 5 min ultrasonication in the presence of 70% ethanol and sterilization under UV lamp for 30 min. The probes were placed in the wells of a 24-well polystyrene plate. Overnight bacterial cultures were dissolved 1:100 in MHB, 500 µL of the suspensions was applied onto the plastic pieces, and the suspension was left for 2 h for completion of the adhesion phase of biofilm formation. The unattached bacteria were then removed, and the wells were gently washed with PBS. Then, the wells were filled with fresh MHB without (control) or with the addition of tobramycin corresponding to half of the MIC determined experimentally for each strain. The samples were incubated for 24 h at 37 °C, the culture media were removed, and the wells were washed. This was followed by fixation for 2 h with 4% glutar aldehyde in Na cacodylate buffer, at a pH of 7.4, 1 h post fixation in 1% cacodylate-buffered OsO_4_, and dehydration in a graded ethanol series. The materials were mounted on sample holders and sputter-coated with gold [36]. The observations were made on a Lyra\Tescan electron microscope (Tescan Analytics, Brno, Czech Republic) at an accelerating voltage of 20 kV.

### 2.13. Statistical Analysis

Quantitative data processing and the preparation of graphs were performed on MS Excel. The data were reported as means ± standard deviation (SD). Comparisons of the differences between the averages were evaluated by one-way ANOVA followed by the Tukey HSD post-estimation test (calculator: https://www.statskingdom.com/180Anova1way.html, accessed on 15 January 2025). Differences were considered statistically significant at *p* ≤ 0.05. Box-plots were prepared using an online calculator (https://www.statskingdom.com/advanced-boxplot-maker.html accessed on 15 January 2025).

## 3. Results and Discussion

### 3.1. Molecular Epidemiological Typing by RAPD-PCR

Molecular typing analyses are essential for characterizing infecting strains from the point of view of their population structure, genetic diversity, and epidemiological relatedness. The typing experiments in this study were performed by RAPD-PCR.

RAPD PCR analysis is generally applied to generate species/strain-specific DNA profiles. Depending on the type of primers and conditions for RAPD-PCR, it is possible to generate adequate amplification profiles through which one can identify new isolates at the level of genus, species, and even strain [37,38]. For CF isolates, these investigations have also contributed greatly to knowledge of the in-host evolution processes [3,39], the identification of transmissible strains [40], etc. Despite some limitations, the RAPD-RCR methodology is considered to have good intra-laboratory reproducibility [15] and has often been used lately in analyses of clinical *P. aeruginosa* strains [41,42,43,44]. These studies have been performed predominantly using primer 272 [31], which was also used in the present study.

The objectives of the molecular typing analyses applied in the current study were to examine the genetic relatedness between the Bulgarian strains isolated prior to and after the inhaled tobramycin cycles and see whether they shared some similarity with some of the CF strains with different geographic origin included in the international reference panel [27]. The external strains chosen for this comparison included five transmissible isolates, with high virulence, morbidity, and/or treatment requirements, and three sets of serial isolates from different stages of patient colonization.

**Figure 1:**
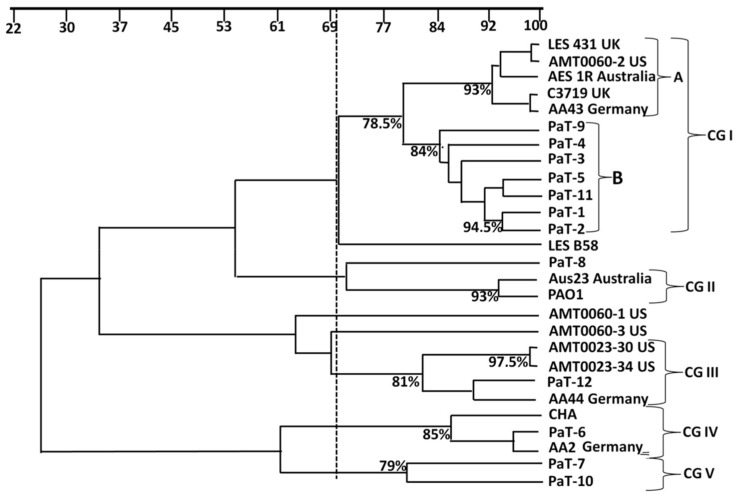
Dendrogram illustrating the relationship between Bulgarian paired *P. aeruginosa* isolates and CF strains of different geographic origin (from the international reference panel [27]) based on analyses with the similarity coefficient (presented as a percentage) of the RAPD profiles generated with primer 272 [31]. The broken line indicates the similarity cut-off value of 70% [32]. CG I to V—cluster groups.

The results were presented as a dendrogram constructed using RAPD data (Figure 1), and the image of the electrophoresis gel is included as Supplementary Figure S1. Using a ≥70% similarity quotient as a cut-off value for defining clonal relatedness, five distinct cluster groups could be differentiated. The largest, Group I, consisted of two subgroups. Subgroup A included five international panel strains with increased morbidity and/or treatment requirements (LES 431, AMT0060-2, AES1R, C3719, and AA43). Subgroup B included seven of our model strains: five pre-treatment (PaT-1, PaT-3, PaT-5, PaT-9, and PaT-11) and two post-treatment (PaT-2 and PaT-4) isolates. The clonal relatedness between the pre-treatment isolates implied common ways of transmission. Unlike the above, most of the post-treatment strains (PaT-6, PaT-8, PaT-10, and PaT-12) were situated far from their pre-treatment counterparts from the same patient. A likely divergence in the present experiment was shown also in two sets of the serially isolated strains from the reference panel: the early isolates AA2 and AMT0060-3 were separated from their corresponding later isolates, resp. AA43 and AA44, and AMT0060-1 and AMT0060-2. Such divergence patterns between sequential isolates could have been due to selection under the combination of host factors during colonization and the antibiotics applied over the course of the disease. In the case of the Bulgarian isolates, the contribution of the tobramycin treatment as a factor of selection pressure, resulting in genotypic divergence, could have therefore been quite probable.

### 3.2. Growth Parameters

Growth parameters are a characteristic used to estimate the fitness of a given strain for a particular environment. In previous studies on CF strains, two cultivation approaches for comparing different isolates have been applied. To examine fitness changes in sequential strains during colonization of CF airways, some researchers have studied the growth parameters in standard laboratory media, usually applying combinations of one rich and one minimal medium, as models of habitats with different nutritional potential [3,14,30]. The other approach is the application of complicated recipes, such as artificial sputum media developed for modeling the milieu in CF lungs [45,46,47,48].

In the present study, the approach with the use of standard laboratory media was applied in order to compare the fitness of the strains isolated prior to and post the inhaled tobramycin treatment of the patients. Since part of our experiments were performed in the presence of tobramycin, MHB was chosen as the rich growth medium. This is the medium of choice recommended by for antibiotic sensitivity testing. The minimal-salt medium used was M63, with 0.04 M glucose as the sole carbon source.

**Figure 2:**
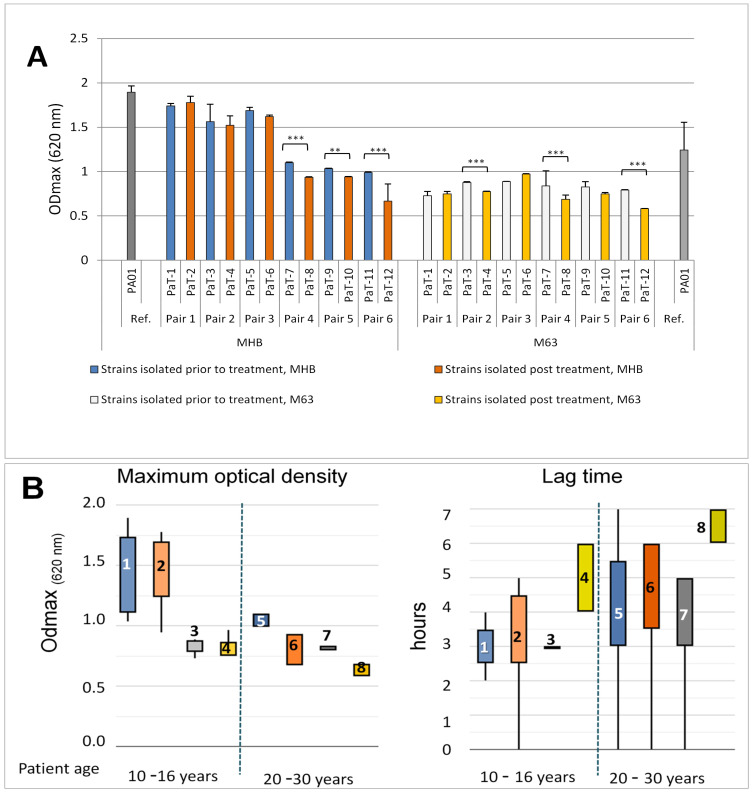
Growth parameters of the strains. (**A**) shows the ODmax values of the strains cultivated in MHB or M63. The one-way ANOVA analysis, with Tuckey’s post-estimation test, revealed a clear-cut distinction between ODmax values within some of the strain pairs, expressed as ** (*p* < 0.01) and *** (*p* < 0.0001). All strains differed significantly from the reference strain PAO1 cultivated in the respective medium. (**B**) ODmax and lag-time duration are presented as box-plots. *P. aeruginosa* isolates were placed in eight groups according to their origin (pre- and post-treatment isolates), the growth media, and the patients’ age: 10–16 years (expected early or intermediate stages of colonization) or 20–30 years (expected late stages of colonization). Legend: (1) MHB, pre-treatment early/intermediate isolate; (2) MHB, post-treatment early/intermediate isolate; (3) M63, pre-treatment early/intermediate isolate; (4) M63, post-treatment early/intermediate isolate; (5) MHB, pre-treatment late isolate; (6) MHB, post-treatment late isolates; (7) M63, pre-treatment late isolate; and (8) M63, post-treatment late isolate. The broken lines separate the data for early/intermediate (10–16 years) and late (20–30 years) isolates.

The growth curve comparisons were focused on the maximal optical density (ODmax) of the bacterial cultures, the growth rate (μ), the doubling time (Td), and the lag-phase duration (Figure 2A; Supplementary Figure S2 and Supplementary Table S1). The comparisons between the ODmax values of the pre- and post-treatment isolates showed a significant variation in this parameter between the strains. Statistically significantly lower values of ODmax for the post-treatment isolates were registered within some pairs; however, such diminution followed no clearly outlined pattern repeating for all strains.

The growth parameter values obtained in the present study were within the range of growth parameters obtained in previous studies for CF isolates, as well as the reference strain, PAO1 [33,46,48]. A similar situation was noted with regard to the lag-phase duration (Figure S2). In both media, an increase in the lag-time was registered for the post-treatment strains compared to their pre-treatment counterparts within the pairs, with the only opposite result for strain couple 3 (PaT-5 and PaT-6). Extension of the lag-phase is generally considered as an adaptive mechanism that may be linked to an increased resistance to stress [49], including the stress response to antibiotics [50].

In the presence of antibacterials, both slow growth and a long lag-phase could provide advantages for the bacteria. One reason for this is that the modes of action of most antibiotics are directed at the molecular mechanisms operating in metabolically active, growing, and dividing bacterial cells. On the other hand, the phenomenon of persistence, i.e., the ability of the bacteria to evade antibiotic killing, has been linked to both their very slow growth and a long lag-phase [51]. The lag-phase is not necessarily functional as a proxy for stress, as, in different cases, it can correlate or not with stress evasion [52]. The mechanisms controlling the lag-phase duration are far from clear. There is some evidence that it can be genetically determined, evolvable, and inheritable, for instance, when there are mutations in the cell-division molecular machinery and/or its regulation [53]. The strains examined here had been maintained as periodically re-cultivated frozen stocks, outside their hosts, in laboratory media, in the absence of antibiotics, for more than 10 years. Therefore, the registration, within five out of the six post-treatment isolates, of longer lag-phases, in comparison to their pre-treatment siblings, indicated changes that were inheritable and confirmed for the two media with different nutritional values.

Other phenotypes that have previously been linked to bacterial growth in CF isolates are the occurrence of small-colony variants and auxotrophy [3,20,32]. Within the present strain collection, the occurrence of small-colony variants on agar plates was registered only for the strains of Pair 2. The presence/absence of bacterial growth in minimal media lacking amino acids is accepted as one of the indicators of auxotrophy [33]. Despite the low ODmax of some strains, all of them grew in the minimal M63 medium, and, therefore, it could be concluded that the hereby examined clinical strains were not auxotrophic.

Previous comparative fitness studies in standard laboratory media on CF strains isolated at different stages of colonization have shown a trend in enhanced fitness of the early isolates compared to the late ones, demonstrated as higher ODmax and growth rate [3,48,54]. The present study included four strain pairs isolated at early or intermediate colonization stages (see Geller, 2009 [23]), i.e., 10–16-year-old patients at the time of isolation (Pairs 1, 2, 3, and 5), and two pairs at late stages of colonization, patient age between 20 and 30 years at the time of strain isolation (Pairs 4 and 6). We plotted the experimental data as box-plots according to the patients’ age at the time of isolation (Figure 2B). The results for the four growth parameters in both the rich medium, MHB, and the minimal M63 medium indicated no clear-cut distinction between the pre- and post-treatment isolates of the early/intermediate patient group. In contrast, the strains isolated from patients over 20 years of age showed a more strict pattern of change in the growth characteristics in the post-treatment isolates (Boxes 6 and 8 in the box-plots, Figure 2B) compared to the pre-treatment strains (Boxes 5 and 7 in all box-plots, Figure 2B), indicating lower ODmax values and longer lag-times.

### 3.3. Biofilm Growth

Biofilm formation is one of the adaptations common for *P. aeruginosa* CF isolates [14,21,22]. They comprise multicellular communities surrounded by extracellular polymeric substances (EPSs) that may be adherent to surfaces or form non-attached multicellular biofilm aggregates [17,55]. While the role of biofilms in chronic and recurrent infections is widely accepted, more recent evidence indicated their participation also in acute bacterial diseases [55,56,57].

Biofilm-associated sessile bacteria are phenotypically and physiologically very different from planktonic cells [58]. Due to the spatial structure of the community, gradients of nutrients and oxygen are generated inside the biofilm. This results in the formation of subpopulations of metabolically active cells situated at the surface, and inward layers of slow-growing or non-growing bacteria [55]. The success of the biofilm mode of life is, however, largely related to the EPS [59]. It contains polysaccharides, extracellular DNA, proteins, and small molecules. The EPS functions as a reservoir for both active biomolecules (e.g., enzymes, diffusible quorum-sensing signals, etc.) and nutrients [59]. Of great importance during CF is the protective role of the EPS against both the defense mechanisms of the host [60] and antibiotics. Polysaccharides have a pivotal role in the protection of sessile bacterial cells. Three types of polysaccharides are released by *P. aeruginosa*: alginate, Psl, and Pel [61,62,63]. The increase in alginate release of the strains during chronification of the infection in CF airways has been pointed out as one of the leading adaptive mechanisms of CF isolates [63]. Alginate has been shown to have various protective functions, among which is the capacity to bind cationic substances like positively charged aminoglycoside antibiotics [61,64,65]. Pel and Psl have also been associated with the neutralization of antibiotics and, hence, biofilm eradication failures [61,66,67]. The interactions of the EPS with antibacterials are, on the one hand, a serious obstacle to infection eradication and, on the other, a prerequisite for the antibiotic tolerance of biofilm bacteria even when derived from a drug-sensitive strain [55,65,68].

**Figure 3:**
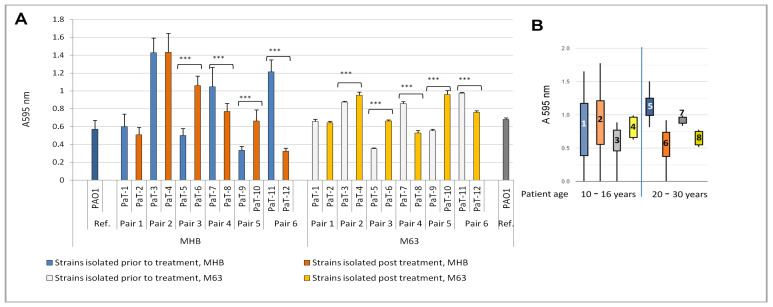
(**A**) Biofilm growth of the strains in MHB or M63 medium. The one-way ANOVA analysis, with Tuckey’s post-estimation test, revealed the strain pairs with clear-cut distinction in biofilm biomass, expressed as *** (*p* < 0.0001). (**B**) The biofilm biomass data are presented as a box-plot. *P. aeruginosa* biofilms were sorted in 8 groups according to their origin (pre- and post-treatment isolates), the growth media, and the patients’ age: 10–16 years (expected early or intermediate stage of colonization) or 20–30 years (expected late stage of colonization). Legend: (1) MHB, pre-treatment early/intermediate isolate; (2) MHB, post-treatment early/intermediate isolate; (3) M63, pre-treatment early/intermediate isolate; (4) M63, post-treatment early/intermediate isolate; (5) MHB, pre-treatment late isolate; (6) MHB, post-treatment late isolate; (7) M63, pre-treatment late isolate; and (8) M63, post-treatment late isolate. The vertical blue line separates the data for early/intermediate (10–16 years) and late (20–30 years) isolates.

The comparison of the biofilm-forming capacity between the strain couples in the present study was performed again in MHB and M63 medium. We aimed to find out whether the inhaled tobramycin treatment had any impact on the in-host changes in this phenotype. The evaluation of biofilm biomass by crystal violet staining, however, produced equivocal results and did not show a distinct pattern of the strain-to-strain differences (Figure 3A; Supplementary Table S2). However, when the data were analyzed separately for early/intermediate and late isolates, it was shown that, in both the rich and minimal media, the post-treatment strains from patients 20–30 years of age produced less biofilm than their pre-treatment counterparts (Figure 3B).

The analysis of the biofilm structure by SEM revealed a variety of morphologies, with no clearly outlined pattern of the differences between pre- and post-treatment isolates (Supplementary Figure S3). Some samples were noticeably more slimy, with most of the cells covered by EPS. Such was the situation with two pre-treatment isolates (PaT-3 and PaT-11) and two post-treatment strains (PaT-2 and PaT-8). Attention was drawn to some apparent differences in cell sizes. For this reason, we performed a morphometric analysis of cell lengths. It showed that most strains were represented by bacteria measuring between 1 and 2 μm. Longer cells, between 2 and 3.5 μm, were registered for two of the post-treatment isolates, PaT-4 and PaT-8 (Supplementary Figure S4). Previous studies have shown that increased lengths of bacterial cells, by increasing the surface area, may provide adaptive advantages in unfavorable environments like those with reduced nutrients, oxidative stress, or the presence of antibiotics [69,70]. This could have also been true for the two post-treatment isolates, PaT-4 and PaT-8.

### 3.4. Invasion and Intracellular Reproduction of the Bacteria in Cultured A549 Cells

Invasion and intracellular reproduction in host cells are mechanisms by which pathogenic bacteria can evade host defense and antibiotic therapy [71]. In spite of the notion that some pathogenic *P. aeruginosa* strains are capable of surviving and reproducing in cultured cells [72], there are only a few studies addressing the occurrence of this phenotype among CF isolates of this species. The complexity of the studies and the lack of standardized laboratory protocols might be among the reasons explaining the small number of studies on invasion by CF strains. The methodological variability includes different types of cultured cells, different amounts of bacteria per cultured eukaryotic cell, i.e., the multiplicity of infection (MOI), the time points for checking intracellular bacteria, the “protection assay”, i.e., the antibiotic applied to avoid bacterial contamination of the external culture medium, etc. [34,73,74,75,76].

In our study, we used the A549 cultured cell line—a human lung adenocarcinoma derived from the peripheral airways of a Caucasian male with lung cancer [77]. This is the most popular model system applied as an in vitro cellular host for *P. aeruginosa* lung isolates [73,76,78]. Next, the concentration of the bacterial suspension during the invasion phase was to be considered. Experimental results in other laboratories with MOI ranging between 100 and 0.1 bacteria per cultured cell have shown that the intracellular bacterial burden is influenced by the MOI during the co-cultivation stage of the experiment, with a higher output at lower levels of MOI, between 1 and 0.1 [75]. We therefore performed our experiments with an MOI of ca. 0.25. Experiments of this type have two stages: co-cultivation of the bacteria with the eukaryotes in the absence of antibiotic, followed by the addition of antibiotic-containing media. Together with the antibiotic–antimycotic supplements standardly applied to DMEM during cell culturing, the bacterial invasion protocols include additional antibiotics to control contamination of the growth medium outside the cells. For this purpose, the “gentamycin protection assay” is usually applied, though some authors choose to use tobramycin [75]. The preliminary invasion experiments in our laboratory, with our model strains, showed that gentamycin did not always prevent extracellular survival of the bacteria. This was in agreement also with the data for the frequent occurrence of gentamycin resistance among CF isolates [33]. We therefore performed our experiments using 100 μg mL^−1^ tobramycin. With this type of protection, the monitoring of extracellular bacterial contamination (by the periodical inoculation of TSA with samples from the DMEM) showed negative growth.

**Figure 4:**
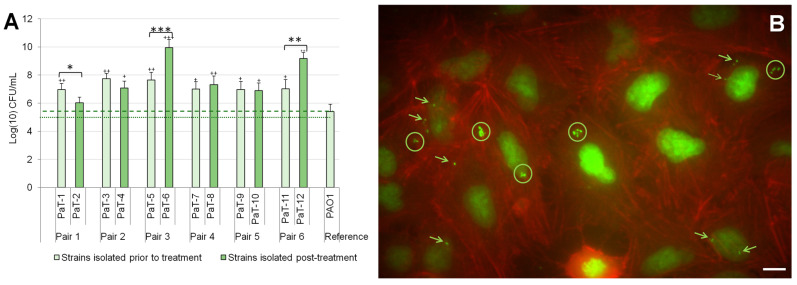
Intracellular growth of the strains in A549 cells. The intracellular bacterial growth is presented as colony-forming units (**A**) and was visualized by fluorescence microscopy (**B**). The dotted line in panel (**A**) stands for the concentrations of the bacterial inoculate, and the dashed line shows the mean invasion value for the reference strain, PAO1. Statistically significant differences (*p* ≤ 0.05) between the CF isolates and the reference strain PAO1 are marked with with + (*p* ≤ 0.05), ++ (*p* ≤ 0.01), or +++ (*p* ≤ 0.001). Significant differences within the strain pairs are labeled with * (*p* ≤ 0.05), ** (*p* ≤ 0.01), or *** (*p* ≤ 0.001). In (**B**), the intracellular location of strain PaT-12 is illustrated. The DNA of both the bacteria and the host cells was colored with SYBR Green, and the actin cytoskeleton was labeled with TRITC-phalloidin and is colored in red. Encircled are intracellular groups of bacteria, and the arrows point to individual bacteria inside the A549 host cells. Scale bar = 10 µm.

The results showed that all the tested CF isolates were capable of intracellular growth and reproduction inside A549 cells (Figure 4A). The intracellular location of the bacteria was confirmed by fluorescence microscopy (Figure 4B). The increase at hour 24 of intracellular bacterial cell counts, compared to the initial bacterial concentration, was within the range of 1 to 5 log units for the model strains. For most strains, except PaT-2, this was significantly higher than the result for the reference strain PAO1. The comparison between the pre- and post-treatment strains varied. For two of the post-treatment strains, PaT-6 and PaT-12, the in vitro results implied a very high capacity for intracellular reproduction. PaT-12 was a late-colonization isolate characterized also by other phenotypic adaptations of late isolates, among which the slow growth resulted in the lowest registered ODmax values in the two tested media. Unlike this, the growth parameters of PaT-6 were within the average of the hereby examined model strains, which did not explain its extreme survivability after 15 inhaled tobramycin cycles (see Table 1). It can be suggested that this strain uses predominantly the intracellular mode of life as a survival strategy to escape both host defense and antibiotics.

### 3.5. Motility

*P. aeruginosa* is capable of three types of motility—the flagella-associated swimming and swarming types of motility and twitching, performed with the participation of Type IV pili [79]. Together with the bacteria’s translocation in space, motility-associated surface appendages have an important role for their adhesion and attachment to surfaces, including mammalian epithelia and basement membranes, and this determines their role in pathogenicity [80]. While these processes provide the pathogen with advantages during its interactions with the host, a major disadvantage for the microorganism is the high immunogenicity of both the flagella and Type IV pili [20]. Therefore, their absence, registered phenotypically as a negative outcome in the motility tests, is considered an advantage of pathogenic *P. aeruginosa*, including CF isolates, for avoiding host recognition [47].

For this reason, tests for the three types of motility were included in the present study. The results showed that all the strains were positive for the flagella-associated motility types, i.e., swimming and swarming (Table 2). The dimensions of the motility zones were within the range observed by other authors [33]. Three of the CF isolates plus the reference strain PAO1 were also positive for twitching motility. The motility data showed no specific trend regarding the isolation time, pre or post treatment.

### 3.6. Tobramycin Susceptibility of the Paired Strains

Tobramycin is the preferred aminoglycoside antibiotic for the treatment of patients with CF infected by *P. aeruginosa* [81,82,83]. It binds to 30S ribosomal subunits and inhibits bacterial protein synthesis [10]. In spite of its effectiveness, studies on serial *P. aeruginosa* strains have pointed to the possibility for the occurrence of variability and changes in the antibiotic sensitivity profiles in sequential isolates from the same patient [15,33].

To check for possible changes in the tobramycin susceptibility of Bulgarian pre- and post-treatment paired isolates, the minimal inhibitory concentrations (MICs) of the strains were determined using graduated test-strips containing a concentration gradient of the antibiotic. The results (Table 3) showed that all the examined clinical isolates from Bulgarian patients with CF were tobramycin-susceptible. Comparing the pre- and post-treatment counterparts within the pairs, we found no MIC change within Pair 1 (strains PaT-1 and PaT-2, with very high genetic similarity, see Figure 1) and Pair 5. For the other four pairs of isolates, some increase in the MIC values in the post-tobramycin treatment strains was registered. Such gradual MIC increase while the strains remain antibiotic-susceptible is known from previous studies on serial isolates [84] and has been linked to the phenomenon of tolerance [85]. Tolerance to antibiotics is distinct from resistance. It enables bacterial survival without the use of conventional resistance mechanisms. While the ge-668 netic clues of resistance have long been in focus of research, the evolution of tolerance is still poorly understood. Up until now, tolerance has been hypothetically linked to genetic mutations leading to increased lag-times and reduced growth rates or biofilm production [54,86].

### 3.7. Phenotypic Shifts in the Strains in the Presence of Sub-MICs of Tobramycin

To check for a possible increase in the tolerance to tobramycin in the survivor strains, we addressed the following question: Are the post-treatment strains more fit than their pre-treatment paired ones when exposed again to the antibiotic? We compared the strains’ fitness parameters (growth, biofilm formation, viability, and motility) during development in the presence of sub-lethal amounts—quarter and half MICs—of the antibiotic (determined for each strain). This phenotypic test was chosen because the CF lung microenvironment is often characterized by sub-lethal amounts of antibiotics. Due to the accumulation of thick mucus, there are gradients in the concentrations of oxygen, nutrients, and other factors. This is certainly also applicable to antibiotics. Therefore, during treatment, bacterial cells can often be subjected to sub-inhibitory amounts of the drugs [28,84,87,88].

**Figure 5:**
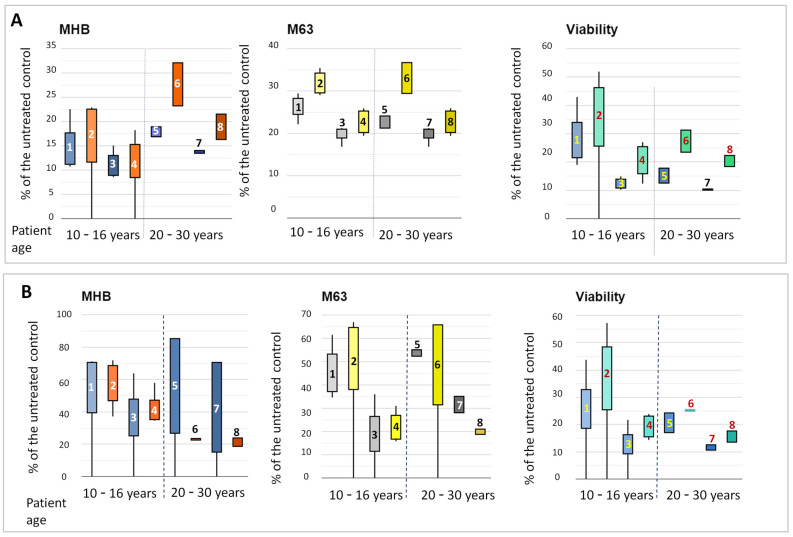
Box-plots of effects of quarter or half doses of tobramycin, determined for each of the strains, on (**A**) bacterial growth in MHB or M63 medium and the viability of the bacterial cells and (**B**) biofilm biomass in MHB or M63 medium and viability of the biofilm cells. All plotted data were calculated as a percentage of the untreated controls of the strains. In both (**A**,**B**), for all tests, the following doses were used: (1) pre-treatment early/intermediate isolates, quarter MIC; (2) post-treatment early/intermediate isolates, quarter MIC; (3) pre-treatment early/intermediate isolates, half MIC; (4) post-treatment early/intermediate isolates, half MIC; (5) pre-treatment late isolates, quarter MIC; (6) post-treatment late isolates, quarter MIC; (7) pre-treatment late isolates, half MIC; and (8) post-treatment late isolates, half MIC. The broken lines separate the data for early/intermediate (10–16 years) and late (20–30 years) isolates.

The values for bacterial growth and biofilm formation and for bacterial viability during cultivation in the presence of the sub-MICs of tobramycin were calculated and presented as a percentage of the corresponding untreated controls (Figure 5 and Supplementary Figures S5 and S6). Figure S5 shows, strain by strain, the relative data for bacterial growth. A trend in better growth of the post-treatment strains (ODmax, as % of the control values) in MHB and M63 media can be seen in Figure S5A,B. The growth capacity of the strains varied for 10 to 30% from that of the untreated controls. Notably, the growth of strain PaT-12 was the least affected. This was the strain with the lowest ODmax values in the two media and the longest lag-phases (see Figure 2 and Supplementary Figure S2). It has previously been shown that some strains with a reduced growth rate produce increased amounts of persister cells [24]. The contribution of persisters to the hereby examined strains with low ODmax and long lag-phases, like PaT-8 or PaT-12, could not be excluded and might have contributed to tobramycin tolerance.

While the effects of sub-MICs on biofilm growth did not follow such a clear-cut trend as the one for broth cultures, it should be underlined that biofilm growth was considerably less affected than in the broth cultures. The values were within the range of 10–80% for MHB-grown biofilm and 10 to 70% for the M63 medium (Supplementary Figure S6A,B). Previous studies have shown that the effects of sub-MICs on biofilm growth can be species- and strain-specific and can vary from reduction to significant enhancement [89]. This also relates to *P. aeruginosa* CF strains, for which both reduction [82] and enhancement [83,87] have been reported.

The bacterial viability in the broth and biofilm cultures was evaluated, relative to the corresponding untreated controls, by the ability of the bacteria to reduce resazurin. This approach has previously been successfully applied in studies on the impact of antibacterial and antibiofilm substances. It has been shown that the estimation of effects by this method provides good correspondence with the CFU counts, and this approach is hence considered reliable for bacterial viability studies [90,91,92]. The present experimental results showed, for both the growth in liquid culture and the biofilm, an even more clearly outlined trend in higher viability of the post-treatment strains in the presence of sub-MICs of tobramycin (Supplementary Figures S5C and S6C).

We then compared the effects of the sub-MICs, taking into account the probable stage of colonization with *P. aeruginosa* (according to [23]). This comparison is presented as box-plots in Figure 5A,B. From these data, it can be suggested that the increase in the tolerance in the post-treatment strains compared to their pre-treatment counterparts (as a % of the untreated controls) was significantly more explicit for growth in broth cultures than for the biofilms. However, the viability change patterns for broth and biofilm were more or less unified and showed a similar trend in better fitness of the post-treatment isolates. This implied that some non-viable bacterial cells contributed to the biofilm biomass evaluated by the crystal violet assay.

**Figure 6:**
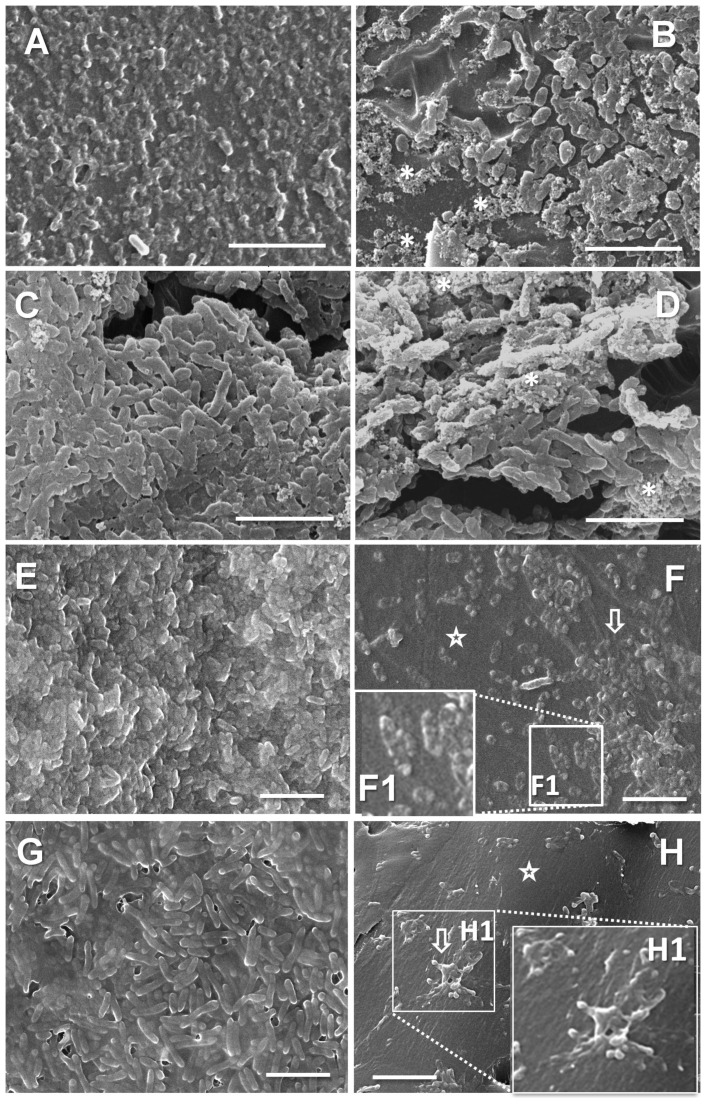
Structural effects of biofilm growth in MHB in the absence (controls (**A**,**C**,**E**,**G**)) or presence of half the MIC of tobramycin (**B**,**D**,**F**,**F1**,**H**,**H1**). (**A**–**D**) are representative images of strains PaT-3 (**A**,**B**) and PaT-4 (**C**,**D**), with comparatively good biofilm growth in the presence of half MIC of the antibiotic, within the range of 60% of the untreated controls. Panels (**E**,**G**) show the untreated biofilms of strains PaT-7 and PaT-8, respectively. These strains grow poorly in the presence of half MIC of the antibiotic and produce biomass within the range of 18–22% of the untreated controls. Legend: asterisks, areas with accumulation of grain-like EPS aggregates; star, biofilm-free area of the substratum; and arrows, pointing to groups of bacteria organized in isles of biofilm. Enlarged area F1 shows details of an expanded blebbed cell. Scale bars = 5 μm.

The suggestion of the presence of altered bacterial cells within the biofilms cultivated in the presence of the sub-MICs was estimated by scanning electron microscopy (SEM). Figure 6 shows images of biofilms—controls or cultivated with a half MIC of tobramycin. Images A to D show strain Pair 2, where the biofilm biomass was the closest to the control for both PaT-3 and PaT-3 (within the range of 60% after the treatment). The images show that the biofilm alterations in these strains, together with a more uneven coverage of the substratum, were also related to the EPS. In the treated samples, the EPS was predominantly represented by grainy aggregates (Figure 6B,D). The other two strains in Figure 6 are PaT-7 and PaT-8 (Pair 4). Unlike Pair 2, these two strains were apparently most affected by the treatment, with a biomass of 14 ± 3% (PaT-7) or 24 ± 3% (PaT-8) relative to the controls. For both these strains, the treated biofilms were organized as separated isles. Some single cells appeared extremely swollen and blebbed—an indication of altered cell envelope integrity (Figure 6F,F1). Other cells were organized in smaller or larger groups, covered by slimy EPS as if in an effort to be self-protected (Figure 6H,H1). Altogether, the results from the SEM investigation confirmed that the biomass of the biofilms developed in the presence of sub-lethal amounts of tobramycin contained both normal cells and bacteria with affected integrity, and the EPS could also be altered.

Bearing in mind the importance of motility for host–bacterial interactions, the effects of the quarter and half MICs of tobramycin on the motility of the strains were also checked (Table 2). It was shown that the swimming motility was completely stopped in all strains. Swarming was generally suppressed, except for PaT-5 and PaT-7, which were motile in the presence of a quarter MIC, and PaT-12, with both sub-inhibitory concentrations. Two of the three strains positive for twitching motility (PaT-2 and PaT-4) retained it in the presence of the antibiotic. The data on the significantly reduced motility did not allow us to outline any pattern related to the time of strain isolation (pre or post treatment). Previous in vitro studies with deletion mutants have shown some co-relation between the deficiency of flagellar proteins and antibiotic tolerance, and it has been suggested that the deficiency in flagellar proteins could be a phenotypic adaptation conferring biofilms increased antibiotic tolerance [93]. To conclude, regardless of whether or not the motility-abolishment response to the sub-MICs of tobramycin in the presently examined strains might have an adaptive role, at this stage, our knowledge is still highly speculative.

## 4. Conclusions

Previous studies on serial *P. aeruginosa* CF isolates have contributed greatly to our understanding of how an opportunistic pathogen can adapt to the specific in-host microenvironment and cause chronic persistent infections [94]. While the data from molecular biology show high levels of genotypic diversification of the bacteria over the course of their within-host evolution, phenotypic studies have revealed the selection of convergent phenotypes, repeatedly occurring in many patients and having adaptive value for CF lung-specific microhabitats [24,95]. The most often described changes in the phenotypic characteristics include slow growth, biofilm formation, increased mucoidity, and reduced motility and virulence [17,24]. Such phenotypic convergence suggests that at least some of these traits are essential for the bacteria to survive under the pressure of factors present inside the host [96]. Together with the specific microenvironment of CF lungs (thick mucus and uneven supply of nutrients and oxygen) and the patient’s immune defense, antibiotic treatment is another pivotal selection factor driving the in-host evolution of bacteria. In spite of this, the studies directed at the impact of antibiotics on the in-host changes in bacteria are sparse. The present study was undertaken with the idea that, if we compared paired strains, isolated from the same patient prior to and post treatment with a given antibiotic, this could provide some clues for understanding the impact of this antibiotic treatment on within-host evolution. On the model of strains of *P. aeruginosa* isolated pre and post inhaled tobramycin treatment, we performed a comparative genotyping (by RAPD-PCR) and phenotyping study. Since multi-trait phenotyping has lately been widely accepted for such studies, we compared several traits: growth parameters, biofilm formation, invasion and intracellular reproduction in A549 cells, and motility. The data on the growth parameters revealed some patterns that allowed us to distinguish between pre- and post-treatment isolates. Four of the post-treatment isolates appeared to be less fit for growth under standard laboratory cultivation conditions, with longer lag-phases and lower values of ODmax than their pre-treatment counterparts. All strains were biofilm-forming, invasive, capable of reproducing inside eukaryotic host cells, and positive for swimming and swarming motility; however, the variations in these phenotypes did not show a pronounced pre- to post-treatment pattern of change. The next question we tried to answer was whether patient treatment influenced the tolerance of the bacteria to tobramycin, the in-host factor which we tried to follow. The experiments performed in the presence of sub-lethal amounts of the antibiotic resulted in a further phenotypic split: they revealed that the growth, biofilm formation, and bacterial viability of the post-treatment isolates were less influenced by the presence of the antibiotic (evaluated as a higher percentage than the untreated controls) than the same phenotypes of the pre-treatment counterparts from the same patient. This implied enhanced tolerance to the antibiotic in the post-treatment isolates. We can therefore suggest that cultivation in the presence of sub-lethal antibiotic doses may be used as a valuable addition to multi-trait phenotyping sets, indicating possible changes in drug tolerance. A limitation of the present study was that, in more than one follow-up sputum culture, the last post-treatment *P. aeruginosa* isolate at the end of the collection period was used. In future studies, the inclusion of more than one post-treatment isolate from a given patient could provide a broader view of the variability in/stability of the observed changes. Future studies using metabolomics would also be desirable.

## Figures and Tables

**Table 1:** Paired *P. aeruginosa* strains from Bulgarian patients with CF used in this study.

Pair	Strain	Patient Age at the Time of Strain Isolation (Years)	Number of Inhaled Tobramycin Cycles
Pair 1	PaT-1	15	0
	PaT-2	16	3
Pair 2	PaT-3	15	0
	PaT-4	16	3
Pair 3	PaT-5	13	0
	PaT-6	16	15
Pair 4	PaT-7	25	0
	PaT-8	27	2
Pair 5	PaT-9	10	0
	PaT-10	11	2
Pair 6	PaT-11	20	0
	PaT-12	21	3

**Table 2:** Diameter of motility zones of the strains (controls) and changes in the motility behavior in the presence of sub-MICs of tobramycin.

Strain	Swimming Motility (cm)			Swarming Motility (cm)			Twitching Motility (cm)		
	Control	Quarter MIC	Half MIC	Control	Quarter MIC	Half MIC	Control	Quarter MIC	Half MIC
PaT-1	2.77 ± 0.08	-	-	0.5 ± 0.08	-	-	-	-	-
PaT-2	2.8 ± 0.29	-	-	0.375 ± 0.09	-	-	1 ± 0	0.15 ± 0.05	-
PaT-3	2.6 ± 0.33	-	-	0.6 ± 0.08	-	-	1.075 ± 0.09	-	-
PaT-4	2.7 ± 0.12	-	-	0.6 ± 0.08	-	-	1.125 ± 0.05	1.125 ± 0.05	0.825 ± 0.12
PaT-5	2.1 ± 0.21	-	-	0.65 ± 0.12	0.4 ± 0.8	-	-	-	-
PaT-6	3.5 ± 0.32	-	-	0.75 ± 0.23	-	-	-	-	-
PaT-7	3.75 ± 0.16	-	-	0.825 ± 0.05	0.6 ± 0.08	-	-	-	-
PaT-8	2.6 ± 0.12	-	-	0.625 ± 0.17	-	-	-	-	-
PaT-9	2.1 ± 0.27	-	-	0.45 ± 0.13	-	-	-	-	-
PaT-10	1.47 ± 0.08	-	-	0.125 ± 0.05	-	-	-	-	-
PaT-11	2.95 ± 0.1	-	-	0.85 ± 0.21	-	-	-	-	-
PaT-12	1.95 ± 0.20	-	-	0.374 ± 0.15	0.275 ± 0.05	0.2 ± 0.08	-	-	-
PA O1	2.5 ± 0.43	-	-	1.25 ± 0.28	-	-	2 ± 0.16	-	-

**Table 3:** Minimum inhibition concentrations of tobramycin.

	Strain	MIC (µg mL^−1^)	Susceptibility
Pair 1	PaT-1	0.25	S
	PaT-2	0.25	S
Pair 2	PaT-3	0.50	S
	PaT-4	0.75	S
Pair 3	PaT-5	0.50	S
	PaT-6	1.00	S
Pair 4	PaT-7	0.38	S
	PaT-8	0.75	S
Pair 5	PaT-9	1.50	S
	PaT-10	1.50	S
Pair 6	PaT-11	0.75	S
	PaT-12	1.50	S
Reference	PAO1	1.5	S

Susceptibility was estimated in accordance with EUCAST breakpoint values for MIC S ≤ 2 µg mL^−1^ R> (https://www.eucast.org/fileadmin/src/media/PDFs/EUCAST_files/Breakpoint_tables/v_15.0_Breakpoint_Tables.pdf, accessed on 20 January 2025).

## Data Availability

The original contributions presented in this study are included in the article/Supplementary Materials. Further inquiries can be directed to the corresponding author.

## Supplementary Materials

The following supporting information can be downloaded at https://www.mdpi.com/article/10.3390/microorganisms13040730/s1: Figure S1—Agarose gel electrophoresis of RAPD-PCR products (primer 272, see Materials and Methods). MM, 100 bp DNA ladder (Canvax Biotech, Saint Louis, MO, USA). (1) PaT-1; (2) PaT-2; (3), PaT-3; (4) PaT-4; (5) PaT-5; (6) PaT-6; (7) PaT-7; (8) PaT-8; (9) PaT-9; (10) PaT-10; (11) Pat-11; (12) PaT-12; (13) PAO1; (14) LES B58; (15) LES 431; (16) C3719; (17) AES 1R; (18) AUS23; (19) AA2; (20) AA43; (21) AA44; (22) AMT0023-30; (23) AMT 23-34; (24) AMT0060-1; (25) AMT0060-2; (26) AMT0060-3; and (27) CHA. Table S1—ODmax (620 nm) of the strains upon growth in MHB or M63 medium. Figure S2—Lag-phases of the strains. Table S2—Biofilm biomass of the strains upon growth in MHB or M63 medium, CV staining, A 595 nm. Figure S3—SEM images of the strains grown for 24 h in MHB. Scale bars = 5 µm. Figure S4—Cell size distribution plot of the strains. The values represent the mean ±SD of 30 measurements per strain (6 randomly chosen cells on 5 separate SEM images). Figure S5—Effects of quarter or half MIC on the growth in MHB (A), M63 (B) or the viability of the bacterial cells (C). The data in (A) and (B) are presented as % of the ODmax values of the untreated control samples. Viability was estimated by the reduction in resazurin (Alamar Blue reagent, Invitrogen, Thermo Fisher Scientific, Waltham, MA, USA) by the tobramycin-subjected bacteria calculated as a percentage of the resazurin reduction by untreated control samples. Figure S6—Effects of quarter or half MIC on the biofilm growth in MHB (A), M63 (B) or the viability of the biofilm bacterial cells (C). The data in (A) and (B) are presented as % of the ODmax values of the untreated control samples. Viability was estimated by the reduction in resazurin (Alamar Blue reagent, Invitrogen, Thermo Fisher Scientific, Waltham, MA, USA) by the tobramycin-subjected biofilms calculated as percentage of the resazurin reduction by untreated control samples.
