# Supplementary material for: Diversification of Pseudomonas aeruginosa After Inhaled Tobramycin Therapy of Cystic Fibrosis Patients: Genotypic and Phenotypic Characteristics of Paired Pre- and Post-Treatment Isolates
Source: Microorganisms. 2025 Mar 24;13(4):730. doi: 10.3390/microorganisms13040730 (PMC12029236; doi:10.3390/microorganisms13040730)
Supplement: Supplementary file 1 [file microorganisms-13-00730-s001.zip › microorganisms-3504765-supplementary.pdf]

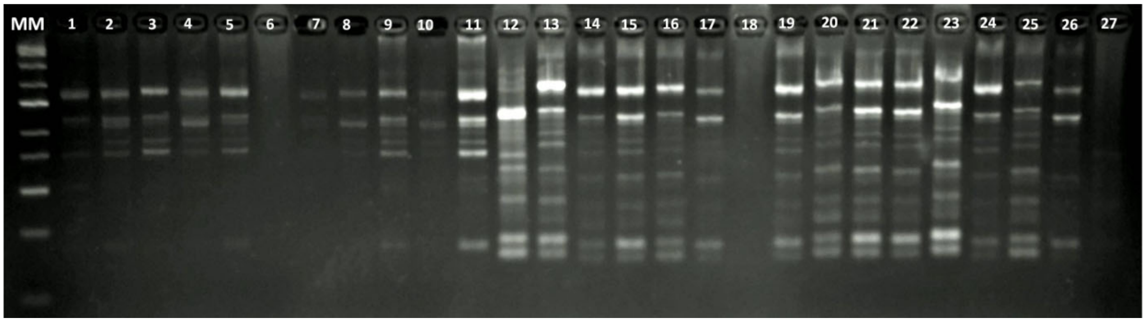

Figure S1. Agarose gel electrophoresis of RAPD-PCR products (primer 272, see Materials and Methods). MM, 100 bp DNA ladder (Canvax Biotech, Saint Louis, MO, USA). (1) PaT-1; (2) PaT-2; (3), PaT-3; (4) PaT-4; (5) PaT-5; (6) PaT-6; (7) PaT-7; (8) PaT-8; (9) PaT-9; (10) PaT-10; (11) Pat-11; (12) PaT-12; (13) PAO1; (14) LES B58; (15) LES 431; (16) C3719; (17) AES 1R; (18) AUS23; (19) AA2; (20) AA43; (21) AA44; (22) AMT0023-30; (23) AMT 23-34; (24) AMT0060-1; (25) AMT0060-2; (26) AMT0060-3; (27) CHA

Table S1. ODmax (620 nm) of the strains upon growth in MHB or M63 medium

		MHB	M63
**Pair 1**	PaT-1	1.74075±0.027729	0.726833±0.047398
	PaT-2	1.77825±0.070325	0.74925±0.025786
**Pair 2**	paT-3	1.56325±0.195948	0.876333±0.008548
	PaT-4	1.52125±0.106628	0.775333±0.003141
**Pair 3**	PaT-5	1.6865±0.03876	0.887167±0.002563
	PaT-6	1.62175±0.016112	0.970667±0.006947
**Pair 4**	PaT-7	1.101±0.007483	0.83775±0.170885
	PaT-8	0.935±0.005477	0.6865±0.048377
**Pair 5**	PaT-9	1.033833±0.002639	0.82725±0.059802
	PaT-10	0.940333±0.004803	0.746667±0.01863
**Pair 6**	PaT-11	0.990167±0.005456	0.790667±0.005574
	PaT-12	0.66775±0.192919	0.581±0.001673
**Reference strain**	PaO1	1.895±0.069649	1.243167±0.312517

The table includes mean±SD of 6 separate measurements per strain

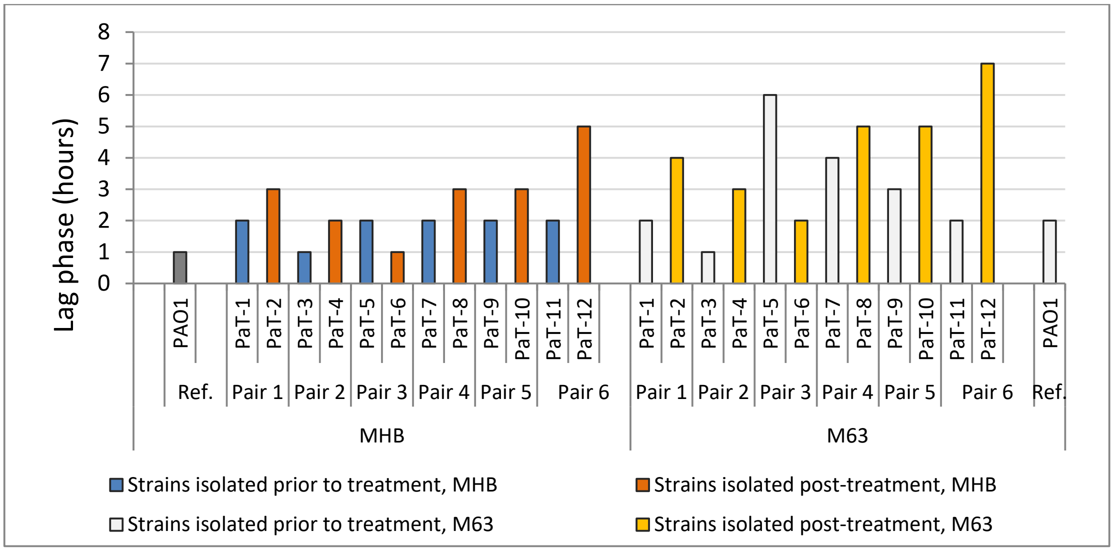

Figure S2. Lag-phases of the strains.

Table S2. Biofilm biomass of the strains upon growth in MHB or M63 medium, CV staining (mean A 595 nm ±SD)

		**MHB (A 595 nm)**	**M63 (A 595 nm)**
**Pair 1**	PaT-1	0.600714±0.139379	0.657333±0.024873
	PaT-2	0.508857±0.081924	0.643333±0.017512
**Pair 2**	paT-3	1.429±0.162274	0.875±0.010488
	PaT-4	1.434111±0.21017	0.953333±0.033267
**Pair 3**	PaT-5	0.501571±0.082395	0.355667±0.005241
	PaT-6	1.060429±0.106781	0.663333±0.012111
**Pair 4**	PaT-7	1.04725±0.215585	0.858333±0.02137
	PaT-8	0.715±0.186921	0.53±0.025298
**Pair 5**	PaT-9	0.3334±0.047016	0.553333±0.017512
	PaT-10	0.6628±0.125724	0.96±0.045166
**Pair 6**	PaT-11	1.2191±0.137647	0.975±0.010488
	PaT-12	0.224429±0.035594	0.76±0.017889
**Reference strain**	PaO1	0.571125±0.100341	0.681667±0.017224

The table includes mean±SD of 6 separate measurements per strain

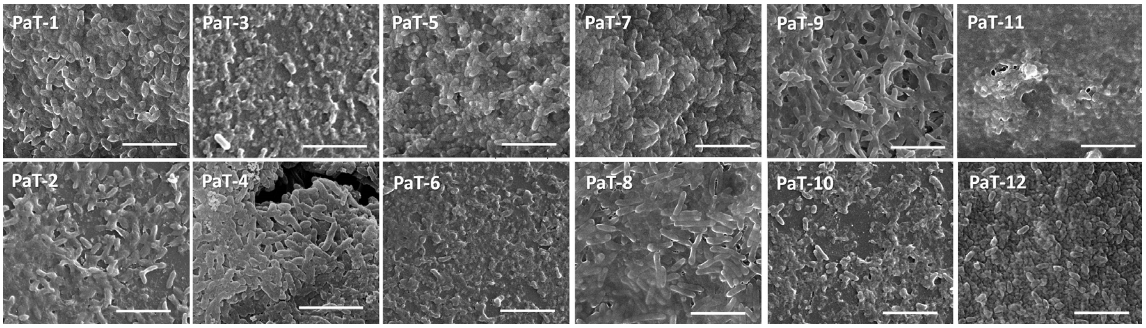

Figure S3. SEM images of biofilms of the strains grown for 24 hours in MHB. Scale bars = 5 µm

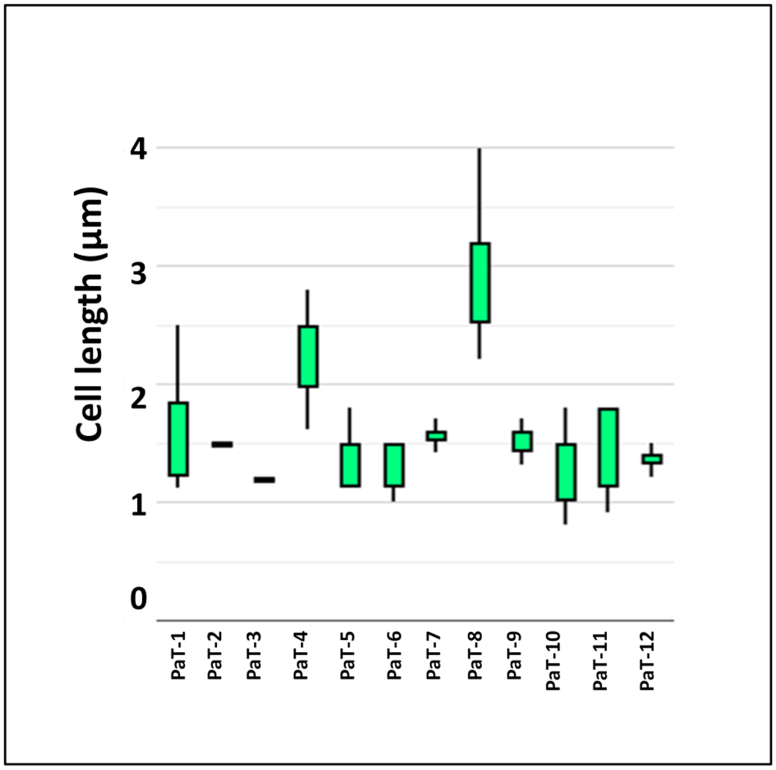

Figure S4. Cell size distribution plot of the strains. The values represent the mean ±SD of 30 measurements per strain (6 randomly chosen cells on 5 separate SEM images)

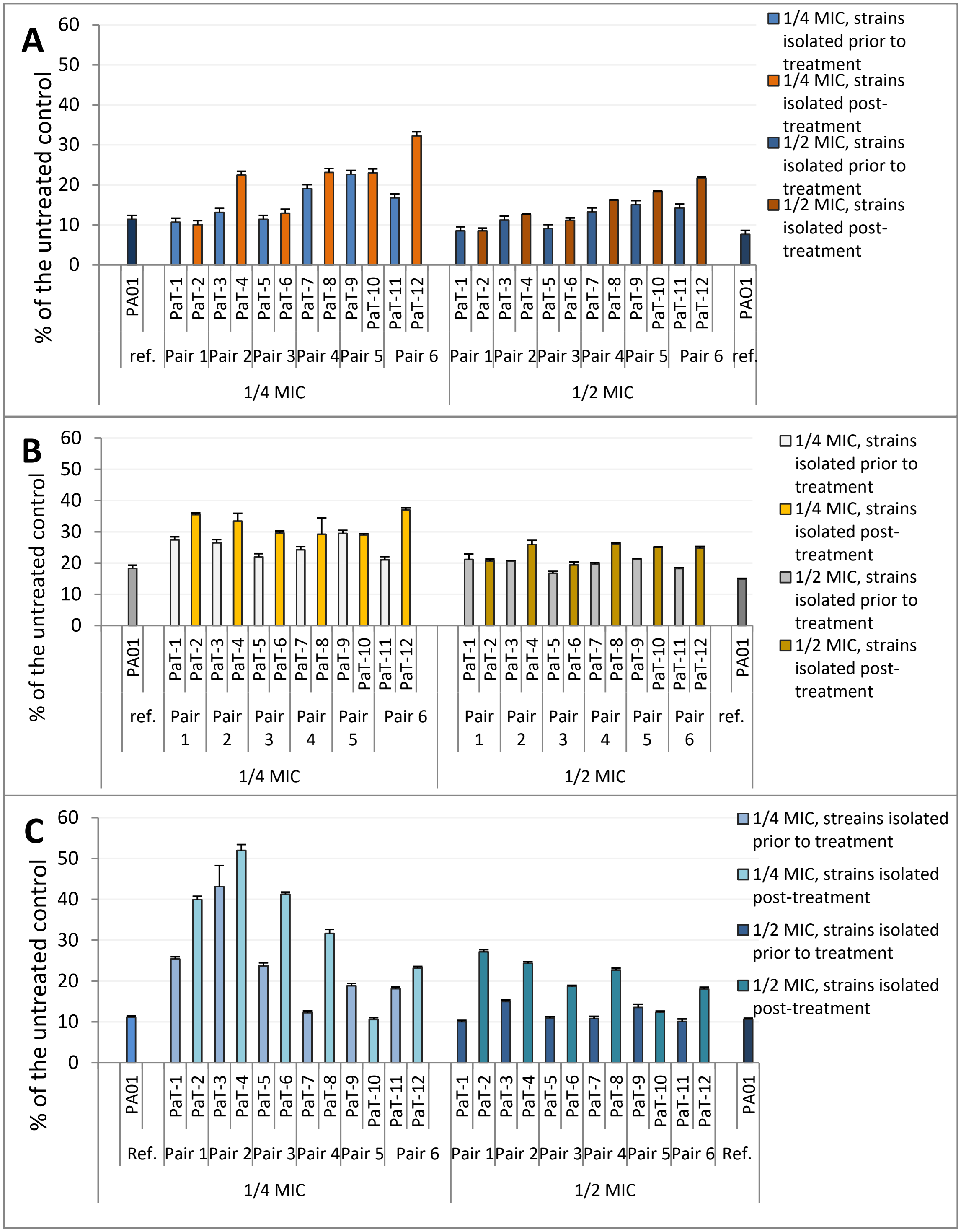

Figure S5. Effects of 1/4 or 1/2 MIC on the growth in (A) MHB, (B) M63, or (C) the viability of the bacterial cells. The data in (A) and (B) are presented as % of the ODmax values of the untreated control samples. Viability was estimated by the reduction of resazurin (Alamar blue reagent, Invitrogen, Thermo Fisher Scientific, Waltham, MA, USA) by the tobramycin-subjected bacteria calculated as per cent of the resazurin reduction by untreated control samples.

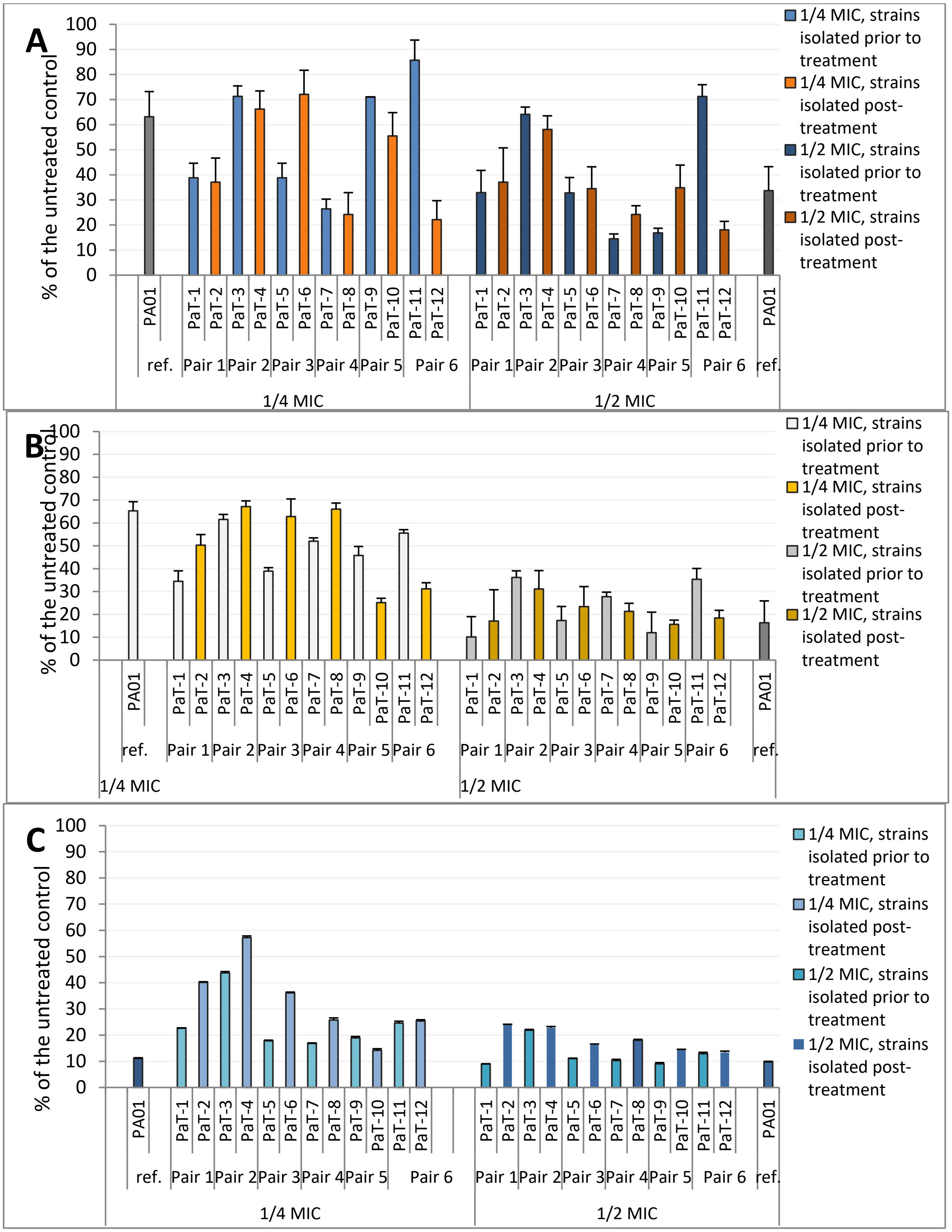

Figure S6. Effects of 1/4 or 1/2 MIC on the biofilm growth in MHB (A),M63 (B) or the viability of the biofilm bacterial cells (C). The data in (A) and (B) are presented as % of the A595 nm values of the untreated control samples. Viability was estimated by the reduction of resazurin (Alamar blue reagent, Invitrogen, Thermo Fisher Scientific, Waltham, MA, USA) by the tobramycin-subjected biofilms calculated as per cent of the resazurin reduction by untreated control samples.
